# Risk score-based substratification improves surveillance costs after transurethral resection of bladder tumor in patients with primary high-risk non-muscle-invasive bladder cancer

**DOI:** 10.1038/s41598-022-17973-8

**Published:** 2022-08-12

**Authors:** Naoki Fujita, Shingo Hatakeyama, Masaki Momota, Yuki Tobisawa, Tohru Yoneyama, Hayato Yamamoto, Hiroyuki Ito, Takahiro Yoneyama, Yasuhiro Hashimoto, Kazuaki Yoshikawa, Chikara Ohyama

**Affiliations:** 1 Department of Urology, Hirosaki University Graduate School of Medicine, 5-Zaifucho, Hirosaki, 036-8562 Japan; 2 Department of Advanced Blood Purification Therapy, Hirosaki University Graduate School of Medicine, 5-Zaifucho, Hirosaki, 036-8562 Japan; 3 Department of Advanced Transplant and Regenerative Medicine, Hirosaki University Graduate School of Medicine, 5-Zaifucho, Hirosaki, 036-8562 Japan; 4 Department of Urology, Aomori Rosai Hospital, 1-Minamigaoka, Shiroganemachi, Hachinohe, 031-8551 Japan; 5 Department of Urology, Mutsu General Hospital, 1-2-8 Kogawamachi, Mutsu, 035-8601 Japan

**Keywords:** Cancer, Urological cancer, Urology, Bladder

## Abstract

High-risk non-muscle-invasive bladder cancer (NMIBC) has a heterogeneity and intensive surveillances after transurethral resection of bladder tumor (TURBT) are major factors of increased costs. Therefore, we aimed to develop optimized surveillance protocols based on the risk score-based substratifications to improve surveillance costs. We retrospectively evaluated 428 patients with primary high-risk NMIBC who underwent TURBT. Patients were substratified into intra-lower, intra-intermediate, and intra-higher groups or UUT-lower, UUT-intermediate, and UUT-higher groups by summing each of the independent risk factors of intravesical and UUT recurrences, respectively. The optimized surveillance protocols that enhance cost-effectiveness were then developed using real incidences of recurrence after TURBT. The 10-year total surveillance costs were compared between the European Association of Urology (EAU) guidelines-based and optimized surveillance protocols. The Kaplan–Meier curves of intravesical and UUT recurrence-free survivals were clearly separated among the substratified groups. The optimized surveillance protocols promoted a 43% reduction ($487,599) in the 10-year total surveillance cost compared to the EAU guidelines-based surveillance protocol. These results suggest that the optimized surveillance protocols based on risk score-based substratifications could potentially reduce over investigation and improve surveillance costs after TURBT in patients with primary high-risk NMIBC.

## Introduction

Bladder cancer (BC) is the ninth most common cancer worldwide^1,2^. BC is well known as one of the most expensive cancers to manage on a per capita basis^3,4^, and it is estimated to account for > 3% of all cancer-related medical costs^5^. Several studies have reported that most costs in patients with non-muscle-invasive BC (NMIBC) are related to surveillance after transurethral resection of bladder tumor (TURBT)^6–8^.

Several guidelines recommend risk-stratified surveillance protocols after TURBT in patients with NMIBC^9–11^. However, because those surveillance protocols were developed based on retrospective studies^12–15^, the optimal surveillance schedules remain indistinct. Because of the heterogeneity in high-risk NMIBC and the fact that risk factors differ between intravesical and upper urinary tract (UUT) recurrences^12,15,16^, we speculated that high-risk NMIBC could be substratified by each of the risk factors of intravesical and UUT recurrences and that the substratification-based surveillance protocols might improve surveillance costs in patients with high-risk NMIBC.

The aim of the present study was to substratify patients with high-risk NMIBC using risk scores calculated by summing each of the independent risk factors of intravesical and UUT recurrences and to develop optimized surveillance protocols based on these substratifications to improve surveillance costs after TURBT in patients with primary high-risk NMIBC.

## Methods

### Ethics statement

This study was performed in accordance with the ethical standards of the Declaration of Helsinki and approved by the Ethics Review Board of Mutsu General Hospital and Aomori Rosai Hospital (authorization numbers: H29-8 and 44, respectively). Pursuant to the provisions of the ethics committee and the ethics guidelines in Japan, a written informed consent was not required for the public disclosure of study information in the case of retrospective and/or observational study using materials, such as the existing documents.

### Patient selection

A total of 480 patients with NMIBC who were treated from November 1993 to April 2019 at Mutsu General Hospital and Aomori Rosai Hospital were evaluated retrospectively. Of the 480 patients, 52 were excluded as they met one or more of the following exclusion criteria: (1) recurrent BC; (2) previous and/or simultaneous UUT urothelial carcinoma (UC); (3) pure carcinoma in situ (CIS) of the bladder; and (4) classified as low- or intermediate-risk based on the European Association of Urology (EAU) guidelines.

### Evaluation of variables

The following variables were analyzed: age; sex; Eastern Cooperative Oncology Group performance status; body mass index; history of hypertension, diabetes mellitus, cardiovascular disease, and chronic kidney disease (CKD); number of tumors; tumor size; pathological T stage; tumor grade; variant histology of UC; lymphovascular invasion (LVI); postoperative intravesical instillation of chemotherapy and bacillus Calmette-Guérin (BCG); and second TURBT. Renal function was evaluated by estimated glomerular filtration rate (eGFR) using a modified version of the abbreviated Modification of Diet in Renal Disease Study formula for Japanese patients^17^ and CKD was defined as eGFR < 60 mL/min/1.73 m^2^. Tumor stage was assigned according to the 2009 TNM classification of the Union of International Cancer Control. Tumor grade was classified according to the 1973 World Health Organization classification system. Tumor grade at first TURBT was used in the analyses.

### Follow-up protocol

Our follow-up protocol was based on the EAU guidelines (urine cytology and cystoscopy every 3 months for 2 years, every 6 months for an additional 3 years, and annually thereafter as well as abdominal and pelvic computed tomography [CT] and blood chemistry to evaluate renal function for contrast-enhanced CT annually; Table 1). Disease recurrence site was classified as intravesical and UUT. The first recurrence in each site after TURBT was recorded.

**Table 1:** Surveillance protocols.

EAU guidelines-based protocol	Months after TURBT																		
	3	6	9	12	15	18	21	24	30	36	42	48	54	60	72	84	96	108	120
**Cystoscopy and urine cytology**																			
High-risk	●	●	●	●	●	●	●	●	●	●	●	●	●	●	●	●	●	●	●
**Computed tomography and blood chemistry**																			
High-risk				●				●		●		●		●	●	●	●	●	●

Risk score-based protocol	Months after TURBT																		
	3	6	9	12	15	18	21	24	30	36	42	48	54	60	72	84	96	108	120
**Cystoscopy and urine cytology**																			
Intra-lower	●	●	●			●	●		●	●	●	●	●	●		●			●
Intra-intermediate	●	●	●	●	●	●	●	●	●	●	●	●	●	●	●		●	●	●
Intra-higher	●	●	●	●	●			●	●				●		●				●
**Computed tomography and blood chemistry**																			
UUT-lower														●					
UUT-intermediate										●	●	●		●		●			
UUT-higher		●	●			●						●							

EAU, European Association of Urology; TURBT, transurethral resection of bladder tumor; UUT, upper urinary tract.

### Substratification

We developed risk score-based substratifications using multivariable Cox proportional hazard regression analyses for intravesical and UUT recurrence-free survival (RFS). The risk scores were calculated by summing each of the independent risk factors of intravesical and UUT recurrences, and patients with high-risk NMIBC were substratified into intra-lower (0 score), intra-intermediate (1 score), and intra-higher (2 or 3 scores) groups or into UUT-lower (0 score), UUT-intermediate (1 score), and UUT-higher (2 scores) groups.

### Optimized surveillance protocols

Using the risk score-based substratifications, optimized surveillance protocols that enhance cost-effectiveness were developed using real incidences of recurrence after TURBT. An intravesical recurrence detection rate ([number of patients with recurrence/number of patients with surveillance] × 100) of ≥ 1% during a certain period indicated that routine surveillance using urine cytology and cystoscopy was necessary in this period. On the other hand, an intravesical recurrence detection rate of < 1% during a certain period indicated that routine surveillance was not necessary in this period. Similarly, an UUT recurrence detection rate of ≥ 1% during a certain period indicated that routine surveillance via CT and blood chemistry was necessary in this period.

### Outcome evaluations

Time to first intravesical and UUT recurrences, estimated surveillance cost per one recurrence detection, and 10-year total surveillance cost using the EAU guidelines-based and optimized surveillance protocols were recorded. To estimate the cost–benefit, surveillance costs for detecting one recurrence were calculated (total surveillance cost in a follow-up period/number of patients with recurrence) using an exchange rate of 100 yen to one US dollar. Medical costs were $45 for urine cytology, $95 for cystoscopy, $267 for CT with contrast media, and $24 for blood chemistry. The cost of prescriptions, medications, and doctor fees were not included herein. The 10-year total surveillance cost was compared between the EAU guidelines-based and optimized surveillance protocols.

### Statistical analysis

Statistical analyses were performed using SPSS version 24.0 (SPSS, Inc., Chicago, IL, USA) and GraphPad Prism 5.03 (GraphPad Software, San Diego, CA, USA). Quantitative variables were expressed as median with interquartile range. RFS was evaluated using the Kaplan–Meier method and compared using the log-rank test. A *P* value of < 0.05 indicated statistical significance.

## Results

### Patients’ backgrounds

The median age of the patients and median follow-up period after TURBT were 72 years and 54 months, respectively (Table 2). Although 90 (21%) patients were treated with postoperative intravesical instillation of BCG, no patient was treated with maintenance BCG therapy. Figure S1 shows our schedule of induction course of BCG therapy.

**Table 2:** Patients’ backgrounds.

	All, n = 428
Age, years	72 (64–79)
Male	342 (80%)
Body mass index, kg/m^2^	23 (21–25)
ECOG PS ≥ 1	59 (14%)
Hypertension	246 (58%)
Diabetes mellitus	72 (17%)
Cardiovascular disease	145 (34%)
Chronic kidney disease	140 (33%)
**Number of tumors**	
Multiple	191 (45%)
**Tumor size**	
≥ 30 mm	83 (19%)
**Pathological T stage**	
pT1	415 (97%)
Concurrent carcinoma in situ	22 (5.1%)
**Tumor grade**	
Grade 3	125 (29%)
Variant histology of urothelial carcinoma	10 (2.3%)
Intravesical instillation of chemotherapy	311 (73%)
Intravesical instillation of BCG	90 (21%)
Second TURBT	41 (9.6%)
Intravesical recurrence	140 (33%)
Upper urinary tract recurrence	22 (5.1%)
**MIBC progression**	29 (6.8%)
Cystectomy after MIBC progression	13 (3.0%)
Radiation therapy after MIBC progression	7 (1.6%)
Cancer-specific mortality	17 (4.0%)
Follow-up period, months	54 (27–95)

All data is presented as n (%) or median (interquartile range).

ECOG PS, Eastern Cooperative Oncology Group performance status; BCG, bacillus Calmette-Guérin; TURBT, transurethral resection of bladder tumor; MIBC, muscle-invasive bladder cancer.

### Substratification

**Figure 1:**
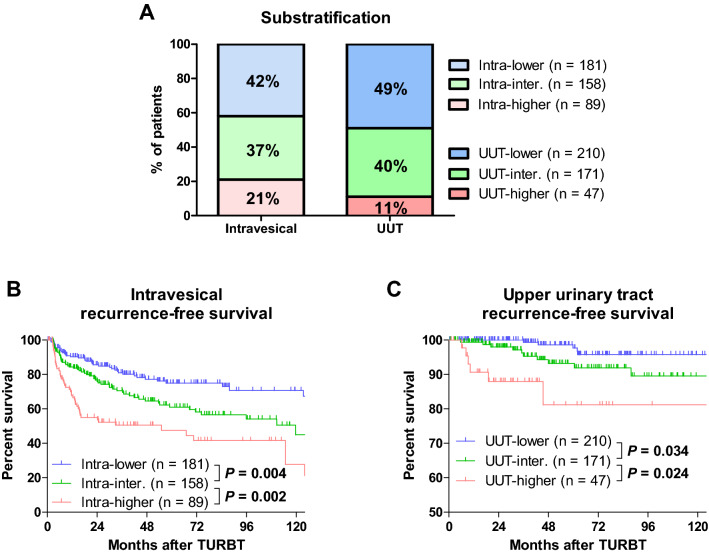
Substratifications and oncological outcomes. Substratifications of high-risk non-muscle-invasive bladder cancer based on risk scores (**A**). Intravesical (**B**) and upper urinary tract (UUT) recurrence-free survival rates (**C**) were evaluated using the Kaplan–Meier method and compared using the log-rank test. TURBT, transurethral resection of bladder tumor.

At the end of the follow-up periods, intravesical and UUT recurrences occurred in 140 and 22 patients, respectively. In multivariable analysis, CKD, grade 3, and tumor size ≥ 30 mm were selected as independent risk factors of shorter intravesical RFS. Similarly, in multivariable analysis, CKD and grade 3 were selected as independent risk factors of shorter UUT RFS (Table 3). Risk scores were calculated by summing each of the independent risk factors of shorter intravesical and UUT RFS (Table 3), and patients with high-risk NMIBC were substratified into intra-lower (0 score, n = 181), intra-intermediate (1 score, n = 158), and intra-higher (2 or 3 scores, n = 89) groups or into UUT-lower (0 score, n = 210), UUT-intermediate (1 score, n = 171), and UUT-higher (2 scores, n = 47) groups (Fig. 1A). The Kaplan–Meier curves of intravesical RFS could be clearly separated among the three groups (Fig. 1B; intra-lower vs. intra-intermediate, *P* = 0.004; intra-intermediate vs. intra-higher, *P* = 0.002). Similarly, the Kaplan–Meier curves of UUT RFS could be clearly separated among the three groups (Fig. 1C; UUT-lower vs. UUT-intermediate, *P* = 0.034; UUT-intermediate vs. UUT-higher, *P* = 0.024).

**Table 3:** Uni- and multivariable analyses for intravesical and upper urinary tract recurrence-free survival.

**Intravesical RFS**	Univariable analyses		Multivariable analysis		Risk score
	*P* value	HR (95% CI)	*P* value	HR (95% CI)	
Age	0.027	1.02 (1.00–1.03)	0.906	1.00 (0.98–1.02)	0
Male	0.992	1.00 (0.66–1.51)			0
ECOG PS ≥ 1	0.010	1.82 (1.16–2.85)	0.233	1.37 (0.82–2.30)	0
Chronic kidney disease	< 0.001	2.19 (1.56–3.07)	0.001	1.99 (1.35–2.94)	1
Multiple tumor	0.432	1.14 (0.82–1.59)			0
Tumor size ≥ 30 mm	0.003	1.78 (1.22–2.56)	0.008	1.67 (1.15–2.42)	1
Pathological T1	0.391	0.72 (0.34–1.53)			0
Concurrent CIS	0.793	1.11 (0.52–2.37)			0
Grade 3	0.005	1.64 (1.16–2.31)	0.030	1.47 (1.04–2.09)	1
Variant histology of urothelial carcinoma	0.171	1.87 (0.76–4.56)			0
Lymphovascular invasion	0.050	2.15 (1.00–4.60)	0.607	1.24 (0.55–2.76)	0
Intravesical instillation of chemotherapy	0.243	0.81 (0.57–1.16)			0
Intravesical instillation of BCG	0.746	1.07 (0.72–1.58)			0
Second TURBT	0.246	0.67 (0.34–1.32)			0

UUT RFS	Univariable analyses		Multivariable analysis		Risk score
	*P* value	HR (95% CI)	*P* value	HR (95% CI)	
Age	0.411	1.02 (0.98–1.06)			0
Male	0.446	1.61 (0.47–5.46)			0
ECOG PS ≥ 1	0.421	0.44 (0.06–3.28)			0
Chronic kidney disease	0.042	2.40 (1.03–5.58)	0.045	2.40 (1.02–5.63)	1
Multiple tumor	0.439	1.40 (0.60–3.30)			0
Tumor size ≥ 30 mm	0.162	1.90 (0.77–4.70)			0
Pathological T1	0.839	0.81 (0.11–6.05)			0
Concurrent CIS	0.254	2.34 (0.54–10.1)			0
Grade 3	0.003	3.69 (1.58–8.65)	0.004	3.53 (1.49–8.33)	1
Variant histology of urothelial carcinoma	0.044	4.47 (1.04–19.2)	0.103	3.42 (0.78–15.0)	0
Lymphovascular invasion	0.423	2.28 (0.30–17.2)			0

RFS, recurrence-free survival; HR, hazard ratio; CI, confidence interval; ECOG PS, Eastern Cooperative Oncology Group performance status; CIS, carcinoma in situ; BCG, bacillus Calmette-Guérin; TURBT, transurethral resection of bladder tumor; UUT, upper urinary tract.

### Time course of recurrence

**Figure 2:**
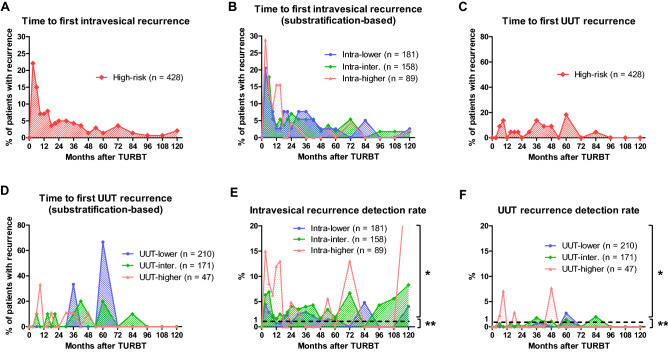
Time courses of recurrence and recurrence detection rates. Time to first intravesical recurrence in all patients with high-risk non-muscle-invasive bladder cancer (NMIBC) (**A**) and in substratified patients (**B**) was evaluated. Time to first upper urinary tract (UUT) recurrence in all patients with high-risk NMIBC (**C**) and in substratified patients (**D**) was evaluated. Intravesical (**E**) and UUT recurrence detection rates (**F**) were evaluated. TURBT, transurethral resection of bladder tumor. *, routine surveillance was needed (≥ 1%). **, routine surveillance was not needed (< 1%).

The first intravesical recurrence occurred most frequently 3 months after TURBT in all patients with high-risk NMIBC (Fig. 2A; n = 31, 22%) and gradually decreased thereafter. Intra-lower and intra-intermediate patients had similar time courses of recurrence (Fig. 2B). Intra-higher patients had significantly more early recurrences within 1 year after TURBT than intra-lower (67% vs. 44%, *P* < 0.001) and intra-intermediate patients (67% vs. 45%, *P* = 0.027). Only 4 (8.9%) recurrences occurred after 30 months in intra-higher patients (Fig. 2B).

The first UUT recurrence occurred throughout the entire follow-up period except after 84 months in all patients with high-risk NMIBC (Fig. 2C). UUT-intermediate patients had a time course similar to that of all patients with high-risk NMIBC (Fig. 2D). No UUT recurrence occurred after 48 months in UUT-higher patients (Fig. 2D).

### Optimized surveillance protocols

Almost all intra-intermediate patients with intravesical recurrence had intravesical recurrence detection rates of ≥ 1% throughout the entire follow-up period, except at 84 months after TURBT (Fig. 2E). On the other hand, intra-higher patients had intravesical recurrence detection rates of < 1% after 30 months, except at 54, 72, and 120 months (Fig. 2E).

UUT-lower, UUT-intermediate, and UUT-higher patients had UUT recurrence detection rates of < 1% throughout the entire follow-up period, except at 60 months (UUT-lower); 36, 42, 48, 60, and 84 months (UUT-intermediate); and 6, 9, 18, and 48 months (UUT-higher) (Fig. 2F).

Based on previously discussed criteria (i.e., a recurrence detection rate of < 1% during a certain period indicated that routine surveillance was not necessary in this period), optimized surveillance protocols were developed to improve surveillance costs (Table 1).

### Economic outcomes

**Figure 3:**
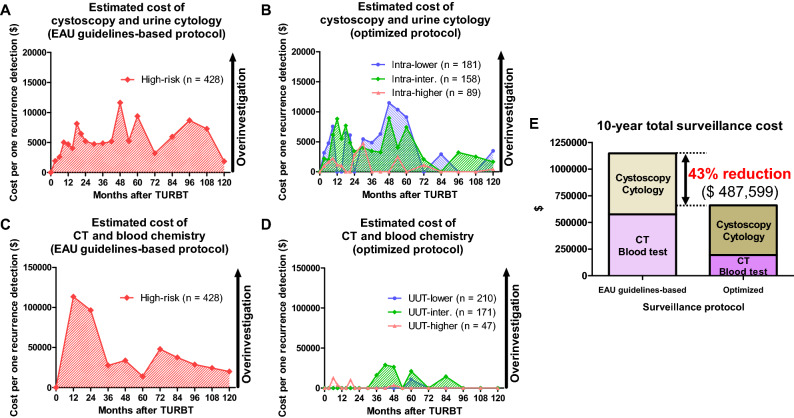
Estimated surveillance costs and 10-year total surveillance cost. Estimated costs of cystoscopy and urine cytology per one recurrence detection (**A**: the European Association of Urology [EAU] guidelines-based surveillance protocol and **B**: the optimized surveillance protocol) were evaluated. Estimated costs of computed tomography (CT) and blood chemistry per one recurrence detection (**C**: the EAU guidelines-based surveillance protocol and **D**: the optimized surveillance protocol) were evaluated. The optimized surveillance protocols promoted a 43% lower ($487,599) 10-year total surveillance cost compared with the EAU guidelines-based protocol (**E**). TURBT, transurethral resection of bladder tumor; UUT, upper urinary tract.

All patients with high-risk NMIBC had a higher (> $5000) estimated cost of cystoscopy and urine cytology per one recurrence detection at 9, 18, 21, 24, 42, 48, 54, 60, 84, 96, and 108 months (Fig. 3A). On the other hand, intra-higher patients had a lower (< $5000) estimated cost of cystoscopy and urine cytology per one recurrence detection throughout the entire follow-up period (Fig. 3B).

All patients with high-risk NMIBC had an extremely high (> $30,000) estimated cost of CT and blood chemistry per one recurrence detection at 12, 24, 48, 72, and 84 months (Fig. 3C). On the other hand, UUT-lower, UUT-intermediate, and UUT-higher patients had lower (< $30,000) estimated costs of CT and blood chemistry per one recurrence detection throughout the entire follow-up period (Fig. 3D).

The optimized surveillance protocols promoted a 43% lower ($487,599) 10-year total surveillance cost compared with the EAU guidelines-based surveillance protocol (Fig. 3E).

**Figure 4:**
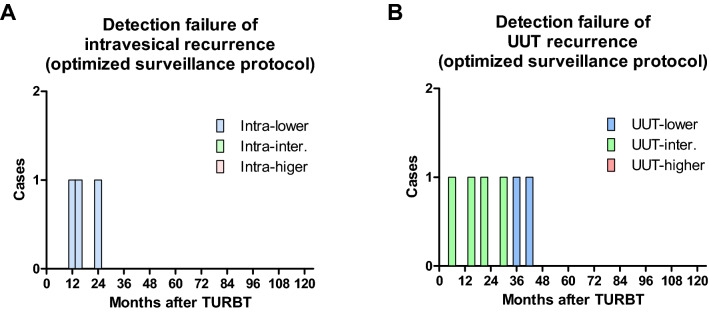
Detection failure of recurrence. The number of patients who potentially failed in intravesical (**A**) and upper urinary tract (UUT) (**B**) recurrence detection using the optimized surveillance protocols. TURBT, transurethral resection of bladder tumor.

Only three intra-lower patients potentially failed intravesical recurrence detection using the optimized surveillance protocol (Fig. 4A). Similarly, two UUT-lower and four UUT-intermediate patients potentially failed UUT recurrence detection using the optimized surveillance protocol (Fig. 4B).

## Discussion

To the best of our knowledge, this is the first study to evaluate the cost-effectiveness of optimized surveillance protocols using risk score-based substratifications in patients with primary high-risk NMIBC. The present study showed that patients with high-risk NMIBC could be substratified by each of the risk factors of intravesical and UUT recurrences, and the optimized surveillance protocols based on the substratifications promoted a 43% reduction in the 10-year total surveillance cost as compared with the EAU guidelines-based surveillance protocol. The key points of cost reduction were the substratification of high-risk NMIBC and the decrease in the frequency of unnecessary cystoscopy, urine cytology, and CT. Although a validation study is needed, optimized surveillance protocols created from risk score-based substratifications might improve surveillance costs after TURBT in patients with primary high-risk NMIBC.

Several guidelines recommend intensive surveillance using cystoscopy and urine cytology after TURBT in patients with high-risk NMIBC^9–11^. Although intensive surveillance may detect recurrences before progression to muscle invasive BC^18^, it results in increased surveillance costs. Strope et al. reported that costs to manage patients with NMIBC have increased since 1993, and this increase was driven by surveillance after TURBT^6^. Likewise, several studies reported that most costs are driven by surveillance using cystoscopy^7,8^. Because the evidence on this topic is lacking, an optimal surveillance protocol that balances oncological benefits with cost-effectiveness remains indistinct. One possible strategy to improve surveillance costs is the substratification of high-risk NMIBC. Because of the heterogeneity of high-risk NMIBC^16,19^, we speculated that it could be substratified by risk factors of intravesical recurrence. Results showed that the Kaplan–Meier curves of intravesical RFS could be clearly separated among intra-lower, intra-intermediate, and intra-higher patients in the present study (Fig. 1B). Moreover, the time course of intravesical recurrence suggests that less frequent surveillance after 30 months might be sufficient to improve surveillance costs for intra-higher patients, considering that almost all recurrences in intra-higher patients occurred within 30 months after TURBT (Fig. 2B, 91%). Liquid biopsy is an alternative potential method for the substratification of high-risk NMIBC and surveillance after TURBT^20–22^. Although liquid biopsy improves the sensitivity and specificity in the detection of recurrence, it may not be used for improving surveillance costs because of its high cost^21,23^. Although it is not easy to develop a “one-size-fits-all” surveillance protocol because of the heterogeneity of high-risk NMIBC and the differences in medical systems among nations, these results may provide the idea that optimized surveillance protocols constructed from risk score-based substratifications can be used to improve surveillance costs in patients with high-risk NMIBC.

Although several guidelines recommend regular UUT imaging after TURBT in patients with high-risk NMIBC^9–11^, no prospective validation study has supported these recommendations. Regular UUT imaging can detect asymptomatic UUT recurrence. However, no study has demonstrated an association between the detection of asymptomatic UUT recurrence and survival benefits. In the present study, cancer-specific survival (CSS) and overall survival (OS) after UUT recurrence were not significantly different between patients with asymptomatic and symptomatic UUT recurrences (Fig. S2A; CSS, *P* = 0.911, Fig. S2B; OS, *P* = 0.555). In addition, most UUT recurrence detection rates were < 1% throughout the entire follow-up period (Fig. 2F). Similarly, Sternberg et al. reported that of 3074 CT examinations, only 15 (0.5%) were able to detect an asymptomatic UUT recurrence^24^. These results suggest that the frequency and duration of regular UUT imaging might need to be reconsidered to balance oncological benefits with cost-effectiveness. Further study is needed to determine the optimal UUT imaging schedules in patients with high-risk NMIBC.

Not only the clinical and pathological factors of tumors but also other clinical factors of patients have a significant impact on intravesical and UUT recurrence after TURBT^25,26^. In the present study, CKD was selected as an independent risk factor of both of intravesical and UUT recurrences and added into risk scores. Several studies support this relationship between CKD and poor oncological outcomes in patients with NMIBC^27–29^. These results suggest that preoperative CKD cannot be ignored in the substratification of patients with high-risk NMIBC. Several possible mechanisms were presumed. One is chronic inflammation and oxidative stress, which are increased in patients with CKD^30^. Oxidative stress activates chronic inflammation and promotes proliferation, carcinogenesis, invasion of tumor cells, angiogenesis, and chemoresistance^31^. Another mechanism is the reduction in DNA repair ability. Reduction in DNA repair ability and chromosomal abnormalities were observed in cells of patients with CKD^32^. The third mechanism is immune dysfunction. The uremic environment induces immune system dysfunction, including macrophage hypoactivity, altered antigen-presenting cell function, increased helper T-cell 1/2 ratio, impaired T-cell activation, and decreased B-cell count^33^. Although no study has investigated the direct association between these mechanisms and NMIBC with CKD, CKD might be useful for substratifying patients with high-risk NMIBC. Further clinical and basic research is needed.

The present study has several limitations. First, this study employed a retrospective design, which prevented us from making definitive conclusions. Second, a relatively small number of patients were enrolled. Third, only 21% of the patients received intravesical instillation of BCG, none of whom received maintenance BCG therapy. In the study using the National Cancer Database that captured data on more than 70% of newly diagnosed cancer cases in the United States, Balakrishnan et al. have reported that of 47,694 patients with high-risk NMIBC, only 24% received BCG therapy regardless guideline recommendations^34^. Thus, the low BCG therapy rate in the present study may reflect a real-world clinical practice. Fourth, only a limited number of patients underwent second TURBT because it is difficult to perform second TURBT in all patients with high-risk NMIBC in clinical practice considering patients’ comorbidities and an operating room capacity. The small number of patients who received intravesical instillation of BCG and underwent second TURBT might cause the early recurrence. Finally, the prolonged study period may have increased the inaccuracy of pathology interpretations.

In conclusion, the optimized surveillance protocols established from risk score-based substratifications could potentially reduce overinvestigation and improve surveillance costs after TURBT in patients with primary high-risk NMIBC.

## Supplementary Information

Supplementary Figures.

## Data Availability

The minimal data set generated during the present study are available from the corresponding author upon reasonable request.
