# Supplementary material for: Risk score-based substratification improves surveillance costs after transurethral resection of bladder tumor in patients with primary high-risk non-muscle-invasive bladder cancer
Source: Sci Rep. 2022 Aug 12;12:13786. doi: 10.1038/s41598-022-17973-8 (PMC9374693; doi:10.1038/s41598-022-17973-8)
Supplement: Supplementary file 1 — Supplementary Figures. [file 41598_2022_17973_MOESM1_ESM.pptx]

## Slide 1
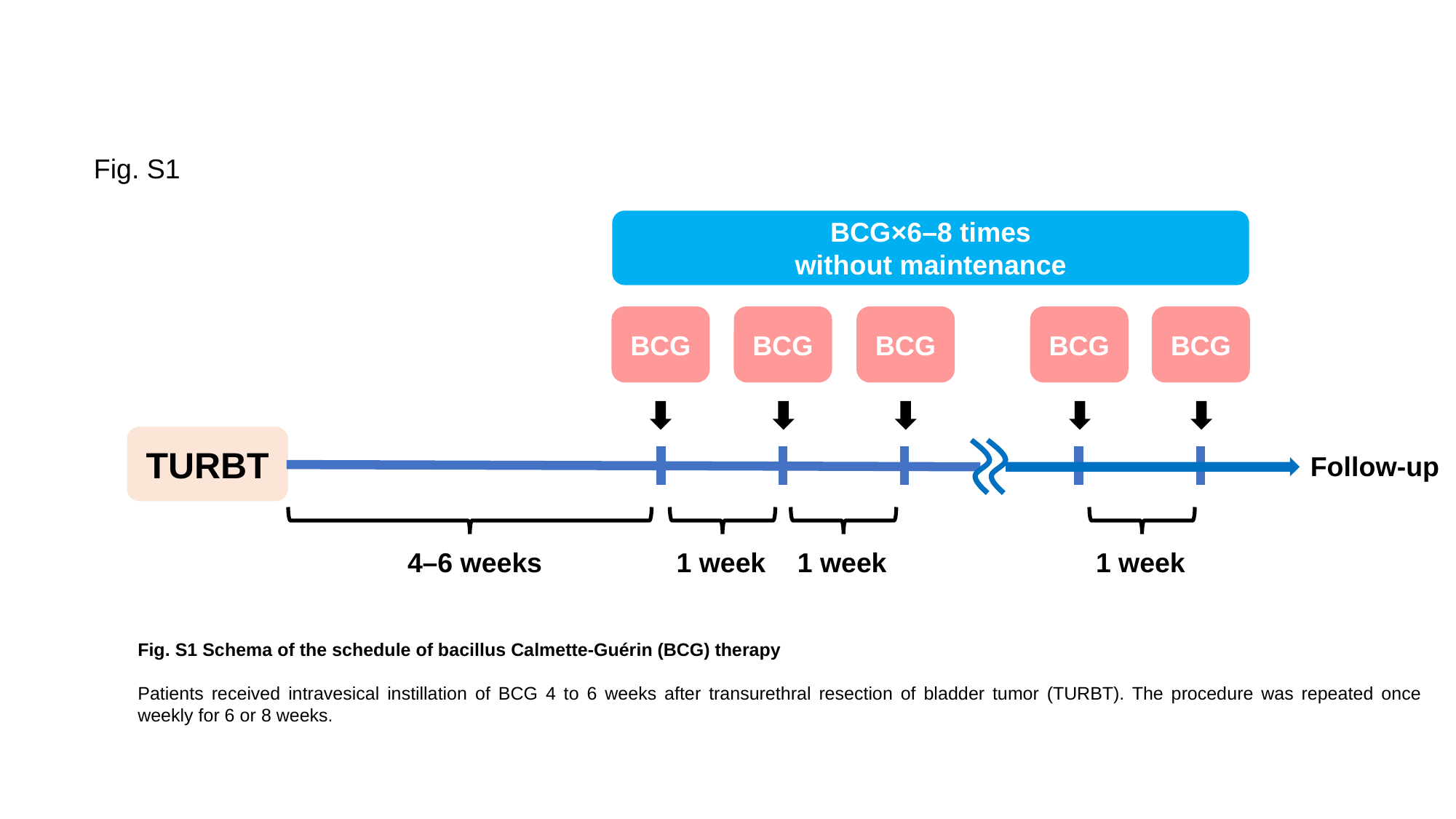

Fig. S1
BCG×6–8 times
without maintenance
BCG
BCG
BCG
BCG
BCG
TURBT
Follow-up
4–6 weeks
1 week
1 week
1 week
Fig. S1 Schema of the schedule of bacillus Calmette-Guérin (BCG) therapy
Patients received intravesical instillation of BCG 4 to 6 weeks after transurethral resection of bladder tumor (TURBT). The procedure was repeated once weekly for 6 or 8 weeks.

## Slide 2
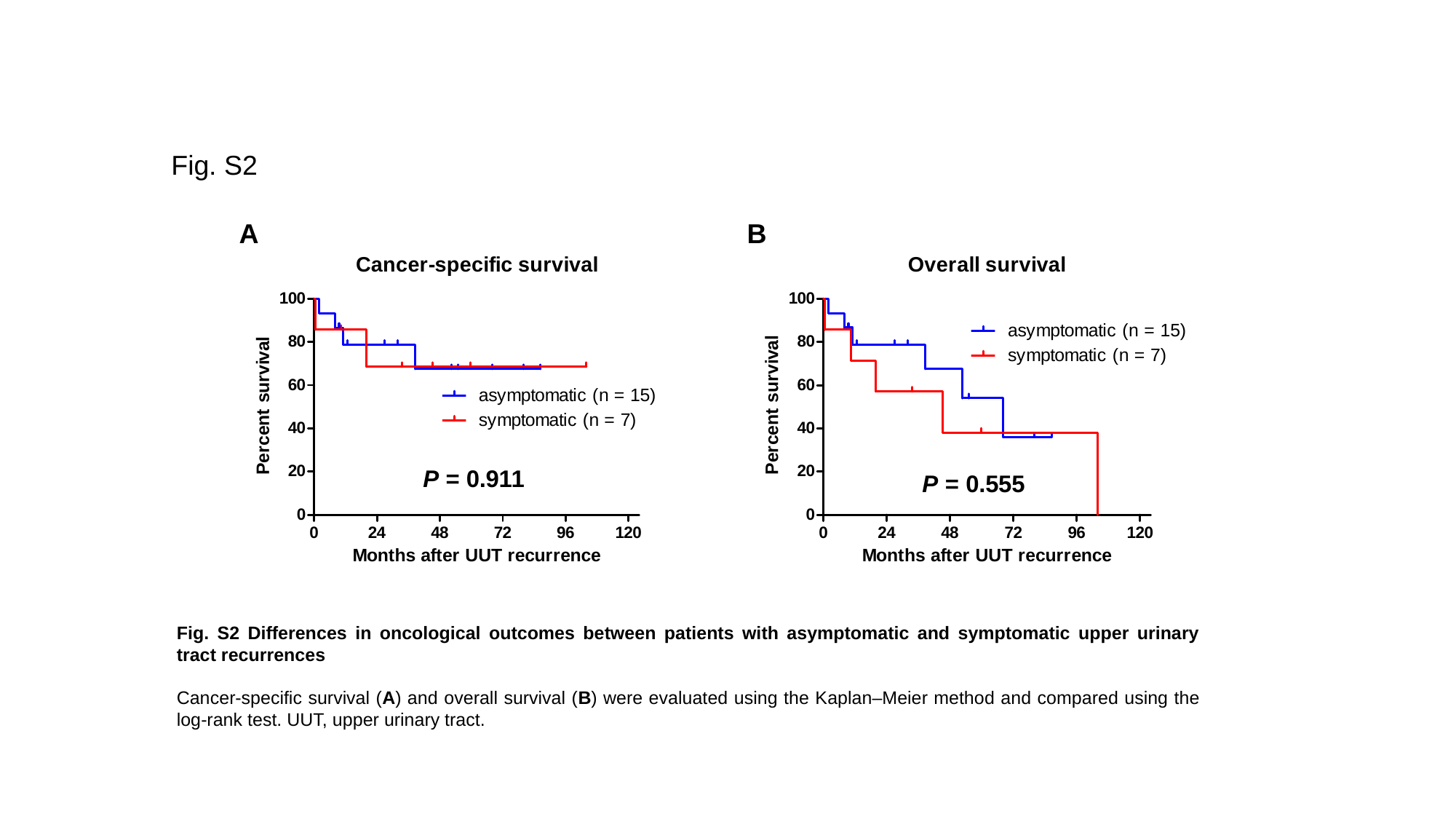

Fig. S2
A
B
Fig. S2 Differences in oncological outcomes between patients with asymptomatic and symptomatic upper urinary tract recurrences
Cancer-specific survival (A) and overall survival (B) were evaluated using the Kaplan–Meier method and compared using the log-rank test. UUT, upper urinary tract.
